# Estimating Chronic Disease Deaths and Hospitalizations Due to Alcohol Use in Canada in 2002: Implications for Policy and Prevention Strategies

**Published:** 2006-09-15

**Authors:** Jürgen Rehm, Norman Giesbrecht, Jayadeep Patra, Michael Roerecke

**Affiliations:** Public Health and Regulatory Policies, Centre for Addiction and Mental Health, University of Toronto. Dr Rehm is also associated with Public Health Sciences, Faculty of Medicine, University of Toronto, Ontario, Canada; the Research Institute for Public Health and Addiction, Zurich, Switzerland; and the Technische Universität Dresden, Germany; Centre for Addiction and Mental Health and Public Health Sciences, University of Toronto, Ontario, Canada; Centre for Addiction and Mental Health and Human Development and Applied Psychology, University of Toronto, Ontario, Canada; Centre for Addiction and Mental Health and Public Health Sciences, University of Toronto, Ontario, Canada

## Abstract

**Introduction:**

Alcohol consumption is a factor that increases risk of chronic disease. This study estimates various indicators of alcohol-attributable premature chronic-disease morbidity and mortality for Canada in 2002.

**Methods:**

Information on mortality and morbidity was obtained from Statistics Canada and from the Canadian Institute for Health Information database. Data on alcohol use were obtained from the Canadian Addiction Survey and weighted for per capita consumption. Risk information was taken from published literature and combined with alcohol consumption information to calculate age- and sex-specific alcohol-attributable chronic disease morbidity and mortality.

**Results:**

In Canada in 2002, there were 1631 chronic disease deaths among adults aged 69 years and younger attributed to alcohol consumption, and these deaths were 2.4% of the deaths in Canada for this age group. The net number of deaths comprised 2577 deaths caused and 947 deaths prevented by alcohol consumption. Moderate drinking was involved in 25% of deaths caused and 85% of deaths prevented by alcohol. There were 42,996 years of life lost prematurely in Canada due to alcohol consumption in 2002, 28,890 for men and 14,106 for women. In Canada in 2002, there were 91,970 net chronic disease hospitalizations attributed to alcohol consumption among individuals aged 69 years and younger. The net numbers were 124,621 hospitalizations caused and 32,651 hospitalizations prevented by alcohol consumption.

**Conclusion:**

With rising rates of alcohol consumption and extensive high-risk drinking, both chronic and acute damage from alcohol are expected to increase. Attention is needed to 1) create effective policies and interventions; 2) control access to alcohol; 3) reduce high-risk drinking; and 4) provide brief interventions for high-risk drinkers.

## Introduction

According to recent studies by the World Health Organization, alcohol consumption is a leading contributor to chronic disease and is recognized as a strong risk factor affecting health in developed countries such as the United States and Canada (1-3). In terms of global burden of disease, about 4% of disability-adjusted life years (DALYs) worldwide in the year 2000 were attributable to alcohol. In developed countries, the percentage is still higher with ranges for men from 8% to 18% and ranges for women 2% to 4% in the Americas and Europe (1). Chronic diseases such as cancer, liver cirrhosis, cardiovascular diseases, neuropsychiatric conditions, and gastrointestinal diseases are affected by alcohol consumption and contribute to global disease burden (3,4). In Canada, aggregate-level studies focused on drinking and disease trends over the past 50 years indicate a strong positive association between average consumption levels and rates of alcohol-attributable mortality (5), liver cirrhosis deaths (6), and total mortality (7).

Both the volume of alcohol consumed and high-risk drinking patterns were found to contribute to chronic disease and disability (8). In this study, the terms *heavy drinking occasion* and *high-risk drinking pattern *are used synonymously with *binge drinking*. High-risk drinking patterns impact chronic diseases, especially ischemic or other cardiovascular disease categories (9). Increased alcohol consumption, as well as increased high-risk drinking, is expected to raise the burden of alcohol-attributable chronic diseases in Canada.

Recorded adult per capita alcohol consumption increased in Canada from 7.3 liters of absolute alcohol per person aged 15 years and older in 1997 to 7.9 liters in 2004 (10,11). According to Canadian Community Health Surveys, high-risk drinking (five or more drinks on one occasion and 12 or more of these occasions in the past 12 months) has increased from about 11.4% in 1994 to 17.8% in 2003 (age-standardized prevalence among the total population aged 15 years and older) (12,13).

Prevention strategies in Canada have not provided adequate attention to the importance of appropriate prevention of alcohol consumption (14). Even when reduction of alcohol consumption is included in prevention strategies, evidence-based effective prevention measures to reduce alcohol-attributable harm, such as higher prices or brief interventions by health care personnel, are often ignored (15).

The general public has little knowledge about the effects of alcohol consumption on chronic diseases (16). Increasing this knowledge should become a public health priority for the following reasons: 1) epidemiological evidence indicates alcohol consumption is a major health risk factor in developed countries (1,3); 2) there is a strong association between recent levels of increased drinking and alcohol-attributable damage in Canada (10,11) and other western countries; 3) damage from alcohol consumption is expected to increase, so it is increasingly important to develop and use evidence-based interventions to reduce alcohol-attributable harm (15); and 4) alcohol tends to be overlooked as a risk factor (17) in chronic disease prevention initiatives in many western countries. This study provides data on the impact of alcohol consumption on chronic diseases by showing estimated premature chronic-disease deaths and hospitalizations caused by alcohol in Canada for the year 2002.

## Methods

### Determining chronic disease categories attributable to alcohol

Chronic disease conditions attributable to alcohol were identified through a review of epidemiological literature that found most assessments converge on the chronic disease categories causally related to alcohol (3,9). This study takes a conservative approach and includes only chronic disease categories with an established biological pathway, temporal order, consistent effects, and dose–response relationships (18). Table 1 gives an overview of chronic conditions identified. The meta-analyses reviewed to determine the nature of the effect of alcohol on chronic conditions in this study showed that the effect of alcohol consumption on chronic diseases can be detrimental or, in some cases, beneficial. The nature of the effect (detrimental or beneficial) of alcohol on chronic conditions depends on the level of alcohol consumed for some conditions, such as stroke (22) or ischemic heart disease (3,21). Lower levels of consumption have a beneficial effect; higher levels of consumption have a detrimental effect. For type 2 diabetes mellitus among men and cholelithiasis (i.e., gall bladder disease) among both sexes, the effect is beneficial for all drinking categories (Table 1). Either detrimental or beneficial effects may be based on confounding phenomena (23), which, in scientific literature, is discussed more for beneficial than for detrimental effects.

### Measurement of alcohol consumption

To measure level of alcohol consumption, we followed the approach of English et al (24) and used four drinking categories based on average volume of alcohol consumed (Table 2). The four levels are 1) abstainer or very light drinker, 0 to less than .25 grams of alcohol per day for men and women; 2) category I, .25 to less than 20 grams of alcohol per day for women and .25 to less than 40 grams for men; 3) category II, 20 to less than 40 grams of alcohol per day for women and 40 to less than 60 grams for men; and 4) category III, 40 grams of alcohol or more per day for women and 60 grams or more for men. Most meta-analyses have given relative risks based on these categories (9).

The prevalence data of different levels of alcohol consumption were collected between 2003 and 2004 through the Canadian Addiction Survey (CAS) (25) with a total sample size of 13,909 (n = 5,721 men and n = 8,188 women) and a 47% response rate. The CAS is a stratified (by region) and randomized (by household and respondent) telephone-based survey of the population aged 15 years and older initiated by the Canadian Centre on Substance Abuse (CCSA). This survey was used despite a relatively low response rate because it had the necessary alcohol consumption measures, a large sample size, and closest temporal proximity to the mortality data provided by Statistics Canada (27). It has previously been reported that higher response rates in surveys did not essentially change distribution results of alcohol consumption (28). 

Characteristics of the CAS sample were weighted to correspond to age and sex distribution of the Canadian population. Average volume of alcohol consumption was derived from a quantity–frequency measure and adjusted by adult per capita consumption (26). Quantity of alcohol was assessed in beverage-specific drinks per usual occasion (25). A per capita consumption adjustment was used because it is considered the most valid choice for alcohol consumption measurements for a population (28). Measurements based on usual quantity and frequency tend to underestimate alcohol consumption because respondents do not usually include heavy drinking occasions in their responses (29).

### Mortality data 

Mortality data, with underlying cause of death coded according to the *International Classification of Diseases, 10th Revision *(*ICD*-10), were obtained from Statistics Canada for 2002 (27). Comparisons of causes of death in the elderly have methodological problems; death certificates tend to be less valid for older individuals than for younger individuals because multiple causes of death are often involved (30,31). In this analysis, only data for those aged 69 years and younger are included because there is indication that relative risks, such as for ischemic heart disease and for substance-attributable mortality and morbidity, tend to converge with increasing age (26) and to result in overestimation of alcohol-attributable deaths caused or prevented in older age groups.

### Computing alcohol-attributable chronic-disease deaths

Alcohol-attributable fractions (AAFs) are generally defined as the proportion of a disease in a population that will disappear if alcohol is removed (32,33). AAFs for this study were calculated from alcohol usage proportions in Canada and disease- and sex-specific pooled relative risks from previous meta-analyses (Table 1) and by using the following formula:

AAF = [Σ^k^_i = 1_P_i_(RR_i _– 1)] /
[Σ^k^_i = 0 _P_i_(RR_i _– 1) +
1]

where i is the category with usage (i = 1) or no alcohol (i = 0), RR(i) is the relative risk at exposure level i compared with no alcohol consumption, P(i) is the prevalence of the i*^th^* category of alcohol consumption, and k is the highest drinking category (i.e., category III).

For each disease category, the sex- and age-group-specific AAFs were calculated as follows: the prevalence of alcohol consumption (exposure category) in the population was multiplied with the excess risk (RR-1) for the given level of alcohol consumption. The numerator in the formula represents the sum of all alcohol-attributable cases of a disease by exposure category within a given sex- and age-group. This sum is divided by all cases of a disease in the given sex- and age-group to derive a proportion that is attributable to the exposure (i.e., alcohol consumption). The counterfactual alternative in this conceptualization is no (or zero) consumption. To derive estimates of the number of deaths due to alcohol for a specific disease (by sex- and age-group), the AAFs were then multiplied by the number of deaths from that specific disease within the sex- and age-group.

We cite a meta-analysis for each condition (e.g., malignant neoplasms, type 2 diabetes mellitus, neuropsychiatric conditions, cardiovascular diseases, digestive diseases) (18) (Table 1) to derive information of relative risk for the diverse categories. Meta-analyses usually cited are from Gutjahr et al (19), a series of meta-analyses based on English et al (24). The Gutjahr et al meta-analyses incorporate literature published since publication of the English et al paper. For some disease categories, other meta-analyses were cited if they were more comprehensive than the Gutjahr et al paper. For stroke, we based relative risks on Reynolds et al (22) because, contrary to other authors, this work separates ischemic and hemorrhagic stroke. Alcohol usage has been shown to differentially influence both types of stroke (3). For hemorrhagic stroke, the effect of alcohol consumption, even at low levels, is detrimental for men (34); for women, the effect may be protective at low levels of drinking (categories I and II) (20), but the effect depends on the pattern of drinking (35). The effect of alcohol consumption on ischemic stroke is partly protective for low to moderate consumption levels (35), and the beneficial effect is more pronounced among women. For depression data, we used mental health surveys to estimate AAFs directly and took into consideration the rates of comorbidity and time of onset for alcohol use disorders and depression in comparison to other mental diseases comorbid with alcohol (3).

No alcohol consumption was used as a counterfactual scenario that was selected for the following reasons: 1) the counterfactual of zero consumption is the most widely used and allows comparisons with other studies; 2) the level of alcohol associated with lowest burden differs from country to country and is different for mortality and morbidity; 3) alcohol-attributable harm is related to average volume of alcohol consumption and consumption patterns; and 4) selection of any volume of consumption other than zero without considering the other dimensions would be arbitrary (36,37).

We calculated AAFs separately by sex and age (men and women 15–29 years, 30–44 years, 45–59 years, 60–69 years). To show detrimental and beneficial effects of alcohol, we applied AAFs to Canadian mortality data (27) to estimate the number of alcohol-attributed deaths by age and sex. A negative AAF, in this case, means that more deaths were prevented than caused by alcohol for the respective disease condition (Table 3).

### Potential Years of Life Lost

We hypothesized that persons dying due to alcohol consumption would have lived longer if they had not consumed alcohol. The average extra time such individuals would have lived is known as *residual life expectancy*. If a man died of alcoholic liver cirrhoses at age 50 in Canada, he would have a residual life expectancy of 28.4 years (38). The sum of residual life expectancies for people dying from alcohol consumption is known as potential years of life lost (PYLL) due to alcohol. PYLL for each sex- and age-group can be estimated by interpolating the observed mean age at death and the standard life expectancies tables for each respective sex– and age–group. This study uses the World Health Organization (WHO) 2000 standard life expectancies table for Canada (38).

To calculate the mean ages within age intervals, we followed rules specified by the WHO Global Burden of Disease study (39). Canadian PYLL were calculated for each age group (15–29, 30–44, 45–59, and 60–69) by multiplying the number of deaths by the interpolated life expectancy for the observed mean age at death for the age interval. The upper age limit of 76.0 years for men and 81.5 years for women was used to approximate Canadian life expectancy at birth. PYLL were calculated per 100,000 population (Table 4).

### Data on hospital diagnoses

Canadian fiscal year 2002 to 2003 hospital diagnoses data (*ICD*-10) were obtained from the Canadian Institute for Health Information (CIHI) Hospital Morbidity Database (HMDB) for both national and provincial levels (40). National data were available for seven provinces (Alberta, British Columbia, Newfoundland, Nova Scotia, Ontario, Prince Edward Island, Saskatchewan) and two territories (Northwest Territories and Yukon) and were provided for disease conditions, and sex- and age-groups in 5-year segments. Data for the provinces of Quebec, Manitoba, New Brunswick, and Nunavut (a northern territory) were not available according to *ICD*-10 classifications. Aggregated data for each condition were estimated based on total population and the combined data of available provinces and territories. The HMDB captures information on patients separated (through discharge or death) from acute-care facilities in Canada and provides national data on acute-care hospitalizations by diagnoses and procedures. Diagnoses were categorized based on most responsible diagnosis (MRD) of hospitalized patients. In cases where multiple diagnoses may be classified as most responsible, the diagnosis associated with the longest stay in a hospital was used. AAFs were then applied to hospital data to estimate alcohol-attributable hospital diagnoses for each treatment facility by age and sex.

## Results

### Alcohol-attributable mortality

This study estimates premature deaths and hospitalizations for Canadians aged 69 years and younger and provides an overview of the 2002 estimated volume of alcohol exposure in Canada by sex- and age-group. As expected, men consumed on average more than women, and alcohol consumption decreased with age.

Table 5 provides estimates of alcohol-attributable deaths — deaths caused or prevented by alcohol consumption. In Canada in 2002, there were 1631 net alcohol-attributable premature deaths estimated, 1155 deaths among men and 476 among women. These numbers were derived by multiplying AAFs with number of deaths for each category to produce numbers with decimals. As a result, there may be minor rounding errors after collapsing numbers across different categories.

The 1631 alcohol-attributable deaths constituted 2.4% of the deaths in Canada for people aged 69 years and younger. These were net figures, and the estimates of deaths prevented by alcohol have been taken into account. There were 2577 deaths (1906 men and 672 women) attributable to alcohol and 947 deaths (751 men and 195 women) prevented by alcohol consumption. Figures were calculated by using the epidemiological procedure previously described.

Moderate drinking (category I, less than 20g per day of pure alcohol for women and less than 40g per day for men) was associated with 25% of deaths caused by alcohol consumption and 85% of deaths prevented by alcohol consumption. In this group of moderate drinkers, the deaths prevented (n = 827) outnumbered deaths caused (n = 677) by alcohol use.

Among premature deaths caused by alcohol, malignant neoplasms accounted for 891 deaths (608 men and 283 women), and digestive diseases accounted for 881 deaths (663 men and 218 women) (Table 3). Among deaths prevented by alcohol for those aged 69 and younger, 788 prevented deaths were ascribed to ischemic heart disease (678 men and 110 women).

**Figure 1:**
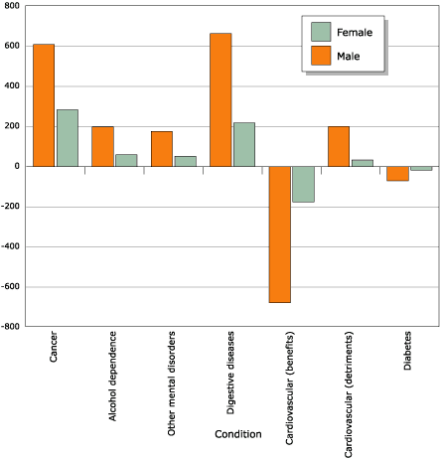
Alcohol-attributable chronic-disease mortality in people aged 69 years or younger in Canada, 2002.

Figure 1 gives an overview of deaths prevented and caused by alcohol. Of the deaths caused by alcohol, including detrimental effects of alcohol on some cardiovascular diseases, cancer (34.6%) and digestive diseases (34.2%) had the highest proportions of deaths caused by alcohol.

**Table:** 

**Condition**	**No. Deaths Caused by Alcohol**		**No. Deaths Prevented by Alcohol**	

	**Men**	**Women**	**Men**	**Women**
Mental disorders (other than alcohol dependence)	237	78	0	0
Digestive diseases	663	218	2	1
Diabetes	0	0	71	18
Cardiovascular	200	33	678	176
Cancer (malignant neoplasms)	608	283	0	0
Alcohol dependence	198	59	0	0
**Total**	**1,906**	**672**	**751**	**195**

### Alcohol-attributable PYLL

In 2002, the PYLL rate for Canada for premature deaths due to alcohol was 196 per 100,000 for men and 92 per 100,000 for women aged 15 to 69 (Table 4). For every population of 100,000, there was a potential net loss of 196 years of life among men and 92 years of life among women as a result of premature death due to alcohol consumption. In total, 42,996 potential years of life were lost due to alcohol in Canada in 2002.

### Alcohol-attributable hospitalizations

Table 6 provides estimates of alcohol-attributable diagnoses for those aged 69 and younger, and Table 7 shows AAFs applied to hospital data to estimate alcohol-attributable fractions and mean age at hospital diagnosis. In Canada for fiscal year 2002 to 2003, 91,970 alcohol-attributable diagnoses among hospital diagnoses from acute care facilities were estimated and accounted for 65,161 hospital separations for men and 26,809 for women. These numbers were derived by multiplying AAFs with number of diagnoses for each category to produce numbers with decimals. As a result, there may be minor rounding errors after collapsing numbers across categories.

**Figure 2:**
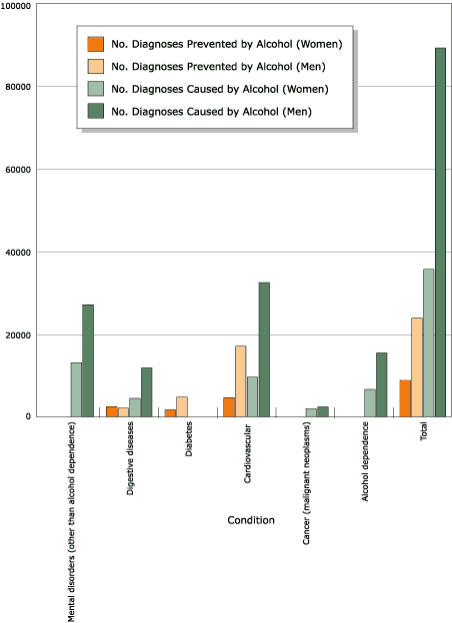
Alcohol-attributable chronic-disease mortality in people aged 69 years or younger in Canada, 2002.

**Table:** 

**Condition**	**No. Diagnoses Caused by Alcohol**		**No. Diagnoses Prevented by Alcohol**	

	**Men**	**Women**	**Men**	**Women**
Mental disorders (other than alcohol dependence)	27,015	13,086	0	0
Digestive diseases	11,803	4,429	2,092	2,458
Diabetes	0	0	4,730	1,698
Cardiovascular	32,422	9,578	17,040	4,633
Cancer (malignant neoplasms)	2,367	1,894	0	0
Alcohol dependence	15,416	6,612	0	0
**Total**	**89,022**	**35,599**	**23,861**	**8,790**

Figure 2 gives an overview of alcohol-attributable chronic disease diagnoses. The number of diagnoses (91,970) is a net figure and includes estimates of hospital separations prevented by alcohol consumption. There were 124,621 acute-care hospital diagnoses (hospital separations for 89,022 men and 35,599 women) caused by alcohol and 32,651 hospital diagnoses (23,861 men and 8790 women) prevented by alcohol consumption. Among acute-care hospital diagnoses attributable to alcohol, neuropsychiatric conditions accounted for 49.9%, cardiovascular disease 33.7%, digestive diseases 13.0%, and cancer (malignant neoplasms) 3.4% of diagnoses (Table 6).

## Discussion

This analysis presents the main associations between alcohol consumption and death and hospitalization due to chronic disease in Canada in 2002. General limitations of the study include the fact that it relies on secondary analysis of existing data, such as official statistics, and these sources have limitations and potential errors. As noted previously, the Canadian Addiction Survey, although it had a modest response rate, was the best resource available to calculate average alcohol consumption by age and sex. People aged 70 and older were excluded from the analysis because there is less confidence in diagnoses among this age group. The assumption that alcohol-attributable relative risks as seen through meta-analyses are transferable between countries may introduce error. Based on meta-analyses used and their systematic exploration of variance, the chronic disease bias may not be large for a country like Canada (24,19,41). 

Another potential bias is that detailed hospitalization data were missing for three provinces and one territory. Because current analyses did not focus on provincial comparisons, the effect is probably minimal. The adjustment for adult per capita alcohol consumption may result in an overestimate because underlying epidemiological estimates were usually not adjusted. In Rehm et al (26), the authors quantified potential overestimation for health effects of alcohol and came to the conclusion that this effect for Canada is limited. Consequently, data are presented with adjustment to be comparable with mainline research such as the WHO Comparative Risk Assessment within the Global Burden of Disease project (1,2,4).

International research, including the WHO Global Burden of Disease study (1), show that alcohol is a risk factor for numerous malignant neoplasms, neuropsychiatric conditions, cardiovascular diseases, and digestive diseases in both developed and developing countries. The number of premature deaths associated with alcohol varies greatly between general categories and specific conditions within a category. Digestive diseases and malignant neoplasms are implicated in similar numbers of alcohol-attributable premature deaths in Canada in 2002, and these disease categories are followed by neuropsychiatric conditions. However, an overall protective effect was estimated for cardiovascular disease mortality.

A somewhat different picture emerges when focusing on hospital diagnoses. The greatest numbers of alcohol-attributable conditions in the hospital diagnoses category are neuropsychiatric conditions associated with alcohol use. These are followed by cardiovascular diseases, digestive diseases, and malignant neoplasms. There are also substantial alcohol-attributable treatments of neuropsychiatric conditions in specialized treatment systems outside of acute-care hospitals.

AAFs generally show at least double the number of deaths or hospitalizations among men compared with women. By age group, the absolute number of deaths is greatest among those aged 45 to 59, followed by those aged 60 to 69. There is a similar age-specific pattern for hospital diagnoses. Total PYLL are estimated at more than 42,000 in Canada for 2002. Alcohol-attributable chronic diseases impact many people during their adult productive years as well as young adults and youth. There are substantial social costs and economic burdens related to diagnoses, treatment, medication, and care.

The findings in this study are generally in line with international research on alcohol and chronic disease as reported in the WHO Global Burden of Disease project (1,2,3) and with analyses of causes of death in the United States (42). In developed countries, such as Canada and the United States, alcohol is a noteworthy risk factor for chronic and acute disease, disability, and death. The WHO Global Burden of Disease project estimated that in developed countries alcohol was responsible for 9.2% of burden of disease compared with 12.2% from tobacco and 10.9% from high blood pressure. The estimated contribution from alcohol was greater than that from high cholesterol, high body mass, low fruit and vegetable intake, or physical inactivity (1,2).

Effective interventions and policies are needed if alcohol-attributable chronic diseases are to be reduced. Babor et al (15) have provided an evidence-based analysis of interventions and alcohol policies that have been shown to be effective in controlling the damage of chronic disease and other harm associated with alcohol use. Their analysis examined hundreds of published evaluations of interventions and rated them on the following four criteria: 1) evidence of effectiveness, 2) breadth of research report, 3) assessment across cultures, and 4) costs to implement. Their analysis took into account the target group in general, adverse side effects, population reach (e.g., number of people affected by the intervention), and political and economic feasibility. The Babor et al assessment pointed to several alcohol-related policies that are effective in controlling and reducing harm from alcohol consumption and that are also relevant in reducing alcohol-related chronic disease. Their study included information on measures that control access to alcohol (e.g., increased real price of alcoholic beverages, lower density of outlets, government alcohol retailing systems), server interventions to prevent service to intoxicated patrons, and brief interventions (short counseling sessions) directed at high-risk drinkers. An increase in real price of alcoholic beverages has been shown to reduce not only the consumption of moderate drinkers but also the consumption of high-risk drinkers as indicated by declining cirrhosis mortality (43). The Babor et al research (15) and supporting work (44–46) indicate that alcohol access control has a preventive impact at the population level for men and women. Access control measures have also been found to have beneficial impacts on specific sectors of a population, such as occasional high-risk drinkers, chronic heavy drinkers, and youth. In general, control measures have greater impact on high-risk drinkers than on moderate drinkers (15,44,46).

Research by Norström based on European and Canadian data (7) has shown that at the population level, cardiovascular mortality levels are not reduced by an increasing rate of alcohol consumption nor elevated by lowering consumption rate. At the population level, there may be no net benefits for cardiovascular disease for those with a higher alcohol consumption level but an increased risk of both chronic and acute damage (7,15).

Additional prevention-based evaluations are needed to assess whether public health benefits of controls on access to alcohol can be further enhanced by combining control measures with other targeted interventions (e.g., server interventions in establishments that sell alcohol and brief interventions for high-risk drinkers who come into contact with health facilities). Population-level interventions could be combined with targeted alcohol awareness messages delivered by health experts and their combined impact assessed. Physicians, nurses, and other health care providers should be encouraged to advise patients routinely about the risks for chronic disease associated with alcohol consumption when they do routine patient monitoring.

Both population-level phenomena (e.g., overall per capita consumption, societal prevalence of high-risk drinking) and individual drinking behaviors are important considerations for alcohol-related prevention initiatives. Efforts to prevent chronic disease need to address risk factors, including alcohol consumption, through focused resources and program coordination in ways that ensure initiatives are informed by epidemiological evidence. Because there is a rising rate of alcohol consumption and high-risk drinking in Canada, increased damage from alcohol is expected. Effective policy, intervention, and prevention efforts are needed, as is public recognition of alcohol as a contributor to chronic disease. Medical, health care, and public health professionals have important roles in drawing attention to alcohol damage and other risk factors for chronic disease and in supporting effective interventions.

## Figures and Tables

**Table 1:** Alcohol-attributable Chronic Disease Categories, Sources for Determining Risk Relations, and Relative Risk (RR), Including Alcohol-attributable Fractions (AAFs), Canada, 2002

Chronic Disease Categories	*ICD*-10 code	Sources for Meta-analyses	Alcohol Consumption Level, RR					

			Drinking Category I^a^		Drinking Category II^b^		Drinking Category III^c^	

			Men	Women	Men	Women	Men	Women
Malignant neoplasms								
Mouth and oropharynx cancer	C00-C14	(19)	1.45	1.45	1.85	1.85	5.39	5.39
Esophageal cancer	C15	(19)	1.07	1.04	1.15	1.08	1.32	1.16
Liver cancer	C22	(19)	1.80	1.80	2.38	2.38	4.36	4.36
Laryngeal cancer	C32	(19)	1.45	1.45	3.03	3.03	3.60	3.60
Breast cancer								
<45 y	C50	(20)	NA	1.15	NA	1.41	NA	1.46
≥45 y	C50	(20)	NA	1.14	NA	1.38	NA	1.62
Other cancers	D00-D48	(3)	1.10	1.10	1.30	1.30	1.70	1.70
Type 2 diabetes mellitus	E10-E14	(19)	0.99	0.92	0.57	0.87	0.73	1.13
Neuropsychiatric conditions								
Alcoholic psychoses	F10.0, F10.3-F10.9	100% AAF^e^	—	—	—	—	—	—
Alcohol abuse	F10.1	100% AAF^e^	—	—	—	—	—	—
Alcohol dependence syndrome	F10.2	100% AAF^e^	—	—	—	—	—	—
Unipolar major depression^d^	F32-F33	(3)	—	—	—	—	—	—
Degeneration of nervous system due to alcohol	G31.2	100% AAF^e^	—	—	—	—	—	—
Epilepsy	G40-G41	(19)	1.23	1.34	7.52	7.22	6.83	7.52
Alcoholic polyneuropathy	G62.1	100% AAFe[Table-fn T1FN5]	—	—	—	—	—	—
Cardiovascular diseases								
Hypertensive disease	I10 - I15	(21)	1.15	1.33	1.53	2.04	2.19	2.91
Ischemic heart disease	I20-I25	(3,21)	0.82	0.82	0.83	0.83	1.00	1.12
Alcoholic cardiomyopathy	I42.6	100% AAF^e^	—	—	—	—	—	—
Cardiac arrhythmias	I47-I49	(19)	1.51	1.51	2.23	2.23	2.23	2.23
Heart failure and ill-defined complications of heart disease^f^	I50-I52, I23, I25.0, I97.0, I97.1, I98.1	—	—	—	—	—	—	—
Cerebrovascular disease	I60-I69	(22)	0.97	0.70	1.08	0.80	1.76	1.96
Ischemic stroke	I60-I62	(22)	0.94	0.66	1.13	0.84	1.19	1.53
Hemorrhagic stroke	I63-I66	(22)	1.12	0.74	1.40	1.04	1.54	1.94
Esophageal varices	I85	(19)	1.26	1.26	9.54	9.54	9.54	9.54
Digestive diseases								
Alcoholic gastritis	K29.2	100% AAF^e^	—	—	—	—	—	—
Cirrhosis of the liver	K70, K74	(3)	1.30	1.30	9.05	9.50	13.00	13.00
Cholelithiasis	K80	(19)	0.82	0.82	0.68	0.68	0.50	0.50
Acute and chronic pancreatitis	K85, K86.1	(21)	1.30	1.30	1.80	1.80	3.20	1.80
Chronic pancreatitis, alcohol induced	K86.0	100% AAF^e^	—	—	—	—	—	—

*ICD*-10 indicates *International Classification of Diseases, 10th Revision*; NA, not applicable

a Drinking category I, .25 to < 20 grams of alcohol per day for women and .25 to < 40 grams of alcohol per day for men.

b Drinking category II, 20 to < 40 grams of alcohol per day for women and 40 to < 60 grams of alcohol per day for men.

c Drinking category III, ≥ 40 grams of alcohol per day for women and ≥ 60 grams of alcohol per day for men.

d For depression, a direct approach was used to estimate AAFs; therefore, RRs are not applicable (3).

e Disease is alcohol-induced; therefore, AAFs are 100%, and RRs are not applicable.

f This unspecific category has no identification of underlying pathology, and the relationship between average volume of alcohol consumption cannot be determined by usual meta-analysis.

**Table 2:** Alcohol Consumption Levels of Respondents to the 2003–2004 Canadian Addiction Survey^a^ (N = 13,909) by Age and Sex^b^

Age Categories, y	Alcohol Consumption Level,^c^ %							

	Abstainer or Very Light Drinker^d^		Drinking Category I^e^		Drinking Category II^f^		Drinking Category III^g^	

	Men	Women	Men	Women	Men	Women	Men	Women
15–29	30.2	59.0	51.6	34.8	8.7	3.2	9.4	3.0
30–44	35.1	62.1	48.6	31.0	8.2	4.3	8.1	2.6
45–59	40.0	65.3	45.5	27.1	7.6	5.5	6.8	2.2
60–69	45.0	68.4	42.4	23.2	7.1	6.6	5.5	1.8
70–79	48.3	70.5	40.4	20.7	6.7	7.4	4.6	1.5
≥80	51.5	72.6	38.3	18.1	6.4	8.2	3.8	1.1
All ages	40.4	66.9	46.8	24.9	6.5	6.3	6.3	1.9

a Source: Canadian Centre on Substance Abuse (25).

b Characteristics of the Canadian Addiction Survey sample were weighted to correspond to age and sex distribution of the Canadian population.

c Average volume of alcohol consumption was derived from a quantity–frequency measure and adjusted by adult per capita consumption (26). Quantity of alcohol was assessed in beverage-specific drinks per usual occasion (25).

d Abstainer or very light drinker category, 0 to < .25 grams of alcohol per day for men and women.

e Drinking category I, .25g to < 20g of alcohol per day for women and .25g to < 40g for men.

f Drinking category II, 20g to < 40g of alcohol for women and 40g to < 60g for men.

g Drinking category III, ≥ 40g of alcohol for women and  ≥ 60g for men.

**Table 3:** Alcohol-attributable Fractions (AAFs)^a^ and Mean Age at Death in Canada, 2002

Chronic Disease Categories	AAF (%) Aged 69 and Younger		Mean Age at Death, y	

	Men	Women	Men (n = 89,022)	Women (n = 35,599)
**Detrimental effects^b^**				
Malignant neoplasms (3.4%)	15.8	4.0	57.3	55.7
Mouth and oropharynx cancer	20.5	8.4	56.9	56.4
Esophageal cancer	19.2	7.1	56.9	57.2
Liver cancer	16.1	7.6	56.9	59.1
Laryngeal cancer	19.3	15.4	59.1	59.1
Breast cancer	0	3.3	0	54.5
Other cancers	3.2	1.0	54.3	53.8
Neuropsychiatric conditions (49.9%)	57.4	35.5	52.1	52.3
Alcoholic psychoses	100	100	51.8	54.4
Alcohol abuse	100	100	53.0	49.9
Alcohol dependence syndrome	100	100	54.7	54.9
Unipolar major depression	1.1	0.1	48.0	54.7
Degenerative nervous system due to alcohol	100	100	59.1	52.0
Epilepsy	39.4	18.7	41.2	46.7
Alcoholic polyneuropathy	100	100	NA	NA
Cardiovascular diseases (33.7%)	2.5	0.3	54.8	56.5
Hypertensive disease	6.4	0.6	56.4	59.7
Alcoholic cardiomyopathy	100	100	52.7	56.4
Cardiac arrhythmias	7.0	1.4	53.7	54.6
Cerebrovascular disease	0.7	NA	56.2	NA
Hemorrhagic stroke	1.4	NA	59.1	NA
Esophageal varices	34.2	17.9	57.5	59.6
Digestive diseases (13.0%)	38.7	55.5	50.3	54.6
Alcoholic gastritis	100	100	58.3	64.5
Cirrhosis of the liver	41.3	26.4	55.6	54.5
Acute and chronic pancreatitis	13.5	3.4	54.4	56.4
Chronic pancreatitis, alcohol induced	55.6	66.7	50.0	52.0
Cholelithiasis	-16.2	-8.6	49.8	42.8
Average mean age at death for detrimental effects			55.2	54.7
**Beneficial effects^b^**				
Type 2 diabetes mellitus	-5.1	-2.5	56.9	57.7
Ischemic heart disease	-3.1	-0.6	57.4	58.6
Cerebrovascular disease	NA	-0.7	NA	56.1
Ischemic stroke	-0.2	-2.9	56.8	53.5
Hemorrhagic stroke	NA	-0.2	NA	59.6
Cholelithiasis	-3.3	-0.8	58.9	54.3
Average mean age at death for beneficial effects			57.4	57.7
Average mean age at death for net effects			53.8	53.4

NA indicates not applicable.

a AAF refers to the number of alcohol-related deaths divided by the overall number of deaths

b Numbers were derived by multiplying AAFs with number of diagnoses and result in numbers with decimals; there may be rounding errors. A negative number indicates that more deaths were prevented than caused by alcohol for the respective disease condition.

**Table 4:** Estimated Potential Years of Life Lost (PYLL) Attributable to Alcohol in Canada, 2002

Age Groups in Years by Sex	Alcohol-attributable Deaths (n)	PYLL
**Men^a^ ^b^**		
15-29	25	1338
30-44	163	6519
45-59	550	14,378
60-69	417	6655
Total for men	1155	28,890
**Women^c^ ^d^**		
15-29	7	402
30-44	75	3349
45-59	235	7191
60-69	160	3164
Total for women	477	14,106
Total	1632	42,996

a Standard life expectancy for men at birth is 76.0 years.

b Alcohol-attributable years of life lost per 100,000 men is 196.

c Standard life expectancy for women at birth is 81.5 years.

d Alcohol-attributable years of life lost per 100,000 women is 92.

**Table 5:** Number^a^ of Chronic Disease Deaths Attributable to Alcohol by Sex, Age, and Disease Category in Canada, 2002

Chronic Disease Categories	No. of Deaths by Age and Sex (N = 2631)							

	15-29 y		30-44 y		45-59 y		60-69 y	

	Men	Women	Men	Women	Men	Women	Men	Women
**Detrimental effects**								
Malignant neoplasms (n = 891)	4	3	35	30	261	124	308	126
Mouth and oropharynx cancer	1	1	11	2	68	11	75	14
Esophageal cancer	1	0	11	2	91	9	105	17
Liver cancer	1	1	9	2	65	15	70	22
Laryngeal cancer	0	0	1	0	31	5	46	8
Breast cancer	NA	1	NA	23	NA	81	NA	61
Other cancers	2	0	3	1	7	3	11	3
Neuropsychiatric conditions (n = 572)	16	3	86	25	188	69	145	40
Alcoholic psychoses	3	0	23	3	41	12	33	8
Alcohol abuse	0	0	15	9	36	14	24	6
Alcohol dependence syndrome	2	0	26	6	92	32	78	21
Unipolar major depression	0	0	0	0	0	0	0	0
Degeneration of nervous system due to alcohol	0	0	0	0	3	1	4	0
Epilepsy	11	3	22	7	16	10	6	5
Alcoholic polyneuropothy	0	0	0	0	0	0	0	0
Cardiovascular diseases (n = 233)	4	1	24	3	88	12	84	18
Hypertensive disease	0	0	4	0	20	2	23	5
Alcoholic cardiomyopathy	0	0	5	1	22	3	8	4
Cardiac arrhythmias	3	1	10	2	26	6	27	7
Cerebrovascular disease	1	NA	5	NA	16	NA	23	NA
Hemorrhagic stroke	0	NA	3	NA	16	NA	35	NA
Esophageal varices	0	0	0	0	3	1	3	1
Digestive diseases (n = 882)	3	1	68	33	320	97	272	88
Alcoholic gastritis	0	0	0	0	1	0	1	1
Cirrhosis of the liver	2	1	62	32	305	93	259	84
Acute and chronic pancreatitis	1	0	3	1	9	2	10	3
Chronic pancreatitis, alcohol induced	0	0	3	0	5	2	2	0
Total detrimental effects (n = 2631)	27	8	213	91	858	301	808	271
**Beneficial effects**								
Type 2 diabetes mellitus	-1	0	-7	-1	-27	-6	-38	-10
Ischemic heart disease	-2	0	-44	-6	-281	-37	-352	-67
Cerebrovascular disease	NA	-1	NA	-8	NA	-23	NA	-34
Ischemic stroke	0	-1	0	-9	-1	-21	-1	-18
Hemorrhagic stroke	NA	0	NA	-1	NA	-4	NA	-9
Cholelithiasis	0	0	0	0	-1	0	-1	0
Total beneficial effects (n = -945)	-2	-1	-50	-16	-308	-67	-390	-111
Total net effects	25	7	163	75	550	235	417	160

NA indicates not applicable.

a Numbers were derived by multiplying alcohol-attributable fractions (AAFs) with number of deaths. Results are in numbers with decimals, and there may be rounding errors. A negative number indicates that more deaths were prevented than caused by alcohol for the respective disease condition.

**Table 6:** Number of Chronic Disease Hospital Diagnoses^a^ by Sex, Age, and Disease Category in Canada, 2002

Chronic Disease Categories	No. of Diagnoses by Age in Years and Sex (N = 124,621)								

	15–29			30–44		45–59		60–69	

	Men	Women		Men	Women	Men	Women	Men	Women
**Detrimental effects**									
Malignant neoplasms (n = 4261)	34	38		216	329	1088	904	1030	623
Mouth and oropharynx cancer	19	15		121	48	437	125	304	85
Esophageal cancer	3	0		30	7	259	40	286	61
Liver cancer	8	3		39	12	248	48	214	61
Laryngeal cancer	2	1		21	5	134	28	210	31
Breast cancer	0	9		0	195	0	564	0	336
Other cancers	1	9		4	62	9	99	16	49
Neuropsychiatric conditions (n = 62,128)	6222	3765		13,358	7069	15,760	6311	7091	2553
Alcoholic psychoses	1690		950	3249	1634	3717	1369	1439	455
Alcohol abuse	1954		1416	3384	1989	3194	1440	1343	477
Alcohol dependence syndrome	1088		689	4524	2448	6554	2438	3249	1037
Unipolar major depression	713		179	1000	281	892	288	344	127
Degenerative nervous system due to alcohol	4		0	33	28	221	62	178	74
Epilepsy	770		531	1157	682	1155	703	516	376
Polyneuropathy	4		0	11	7	26	11	22	7
Cardiovascular diseases (n = 42,000)	666		262	2990	944	13,173	3460	15,592	4913
Hypertensive disease	295		76	1805	491	8480	2129	9453	2752
Alcoholic cardiomyopathy	4		0	77	7	262	32	222	28
Cardiac arrhythmia	320		178	934	416	3768	1190	5386	2051
Cerebrovascular disease	24		NA	76	NA	281	NA	352	NA
Hemorrhagic stroke	19		NA	77	NA	399	NA	615	NA
Esophageal varices	23		8	98	30	382	109	180	81
Digestive diseases (n = 16,232)	516		291	2713	1090	5714	1899	2859	1149
Alcoholic gastritis	136		65	485	195	523	207	213	68
Cirrhosis of the liver	93		51	1108	493	3702	1163	2067	841
Acute and chronic pancreatitis	239		150	688	274	939	377	416	194
Chronic pancreatitis, alcohol induced	48		25	432	128	580	152	163	46
Total detrimental effects (n = 124,621)	7438		4355	19,277	9432	35,735	12,574	26,572	9238
**Beneficial effects**									
Type 2 diabetes mellitus	-229		-111	-629	-252	-1958	-642	-1914	-694
Ischemic heart disease	-46		-13	-1379	-238	-8052	-1420	-7563	-1916
Cerebrovascular	NA		-48	NA	-132	NA	-378	NA	-489
Ischemic stroke	0		-23	-2	-61	-7	-124	-8	-96
Hemorrhagic stroke	NA		-14	NA	-34	NA	-113	NA	-160
Cholelithiasis	-157		-547	-492	-752	-843	-763	-600	-398
Total beneficial effects (n = 32,651)	-432		-719	-2500	-1373	-10,853	-3203	-10,077	-3495
Total net effects (n = 91,970)	7006		3637	16,777	8059	24,883	9371	16,495	5743

a Diagnoses data were obtained from the Hospital Morbidity Database at the Canadian Institute of Health Information (40). Aggregated data for each condition was estimated based on total population and combined data of available provinces and territories. (Provinces included are Alberta, British Columbia, Newfoundland, Nova Scotia, Ontario, Prince Edward Island, and Saskatchewan. Territories included are Yukon and Northwest Territories. *ICD*-10 data were unavailable for Quebec, Manitoba, New Brunswick, and Nunavut.)

**Table 7:** Alcohol-attributable Fractions (AAFs)^a^ and Mean Age at Chronic Disease Hospital Diagnosis^b^ in Canada, 2002

**Chronic Disease Categories**	**AAF (%) All Ages**		**Mean Age at Diagnosis, y**	

	**Men**	**Women**	**Men**	**Women**
**Detrimental effects**				
Malignant neoplasms total	35.2	8.3	55.6	52.9
Mouth and oropharynx cancer^c^	34.9	20.5	53.6	51.7
Esophageal cancer	39.8	26.0	57.2	58.1
Liver cancer	31.6	21.0	55.6	55.8
Laryngeal cancer	44.7	31.7	58.1	56.4
Breast cancer	0	6.7	0	60.7
Other cancers	9.9	5.6	55.9	49.3
Neuropsychiatric conditions total	60.2	29.8	45	42.5
Alcoholic psychosis	100	100	43.9	41.3
Alcohol dependence syndrome	100	100	48.1	45.3
Alcohol abuse	100	100	42.6	39.5
Unipolar major depression	11.7	2.1	41.1	42.9
Degenerative nervous system due to alcohol	100	100	56.7	55.1
Epilepsy	38.4	27.2	42.5	42.6
Alcoholic polyneuropathy	100	100	52.1	51.4
Cardiovascular diseases total	21.9	8.9	56	56.1
Hypertensive disease	25.6	8.5	56.1	56.5
Alcoholic cardiomyopathy	100	100	54.7	55.6
Cardiac arrhythmias	27.7	18.1	56.2	55.7
Cerebrovascular disease	3.8	NA	55.5	NA
Hemorrhagic stroke	10.2	NA	57.4	NA
Esophageal varices	55.4	39.8	52.1	53.4
Digestive diseases total	51.5	30.1	50.3	49.6
Alcoholic gastritis	100	100	45.6	44.5
Cirrhosis of liver	60.1	44.4	52.9	52.6
Acute and chronic pancreatitis	26	12.3	46.6	45.8
Chronic pancreatitis (alcohol-induced)	100	100	47.1	46
Average mean age at diagnosis for detrimental alcohol-related effects			50.0	47.6
**Beneficial effects**				
Type 2 diabetes mellitus	-9.4	-5.2	32.2	26.0
Ischemic heart disease	-10.2	-5.6	56.3	57.6
Cerebrovascular	NA	-7.3	NA	54.6
Ischemic stroke	-0.5	-9.4	55.9	50.7
Hemorrhagic stroke	NA	-4.6	NA	55.3
Cholelithiasis	-16.2	-8.6	49.8	42.8
Average mean age at diagnosis for beneficial alcohol-related effects			48.7	43.5

NA indicates not applicable.

a Alcohol-attributable fractions (AAFs) refer to number of alcohol-related diagnoses divided by overall number of diagnoses.

b Diagnoses data were obtained from the Hospital Morbidity Database at the Canadian Institute of Health Information (40). Aggregated data for each condition was estimated based on total population and combined data of available provinces and territories. Provinces included are Alberta, British Columbia, Newfoundland, Nova Scotia, Ontario, Prince Edward Island, and Saskatchewan. Territories included are Yukon and Northwest Territories. *ICD*-10 data were unavailable for Quebec, Manitoba, New Brunswick, and Nunavut.

c Numbers were derived by multiplying AAFs with number of diagnoses; results are in numbers with decimals, and there may be rounding errors. A negative number indicates that more hospital diagnoses were prevented than caused by alcohol for the respective disease condition.
